# Role of IL-22 in persistent allergic airway diseases caused by house dust mite: a pilot study

**DOI:** 10.1186/s12890-021-01410-z

**Published:** 2021-01-21

**Authors:** Laura Tamasauskiene, Vilte Marija Gintauskiene, Daina Bastyte, Brigita Sitkauskiene

**Affiliations:** Department of Immunology and Allergology, Lithuanian University of Health Sciences, Eiveniu str. 2, 50009 Kaunas, Lithuania

**Keywords:** Persistent allergic airway diseases, Allergic asthma, Allergic rhinitis, House dust mite, IL-22

## Abstract

**Background:**

Persistent allergic airway diseases cause a great burden worldwide. Their pathogenesis is not clear enough. There is evidence that one of the recently described cytokine interleukin (IL) 22 may be involved in the pathogenesis of these diseases. Scientists argue if this cytokine acts as proinflammatory or anti-inflammatory agent. The aim of this study was to investigate IL-22 level in patients with persistent allergic airway diseases caused by house dust mite (HDM) in comparison with healthy individuals and to evaluate its relationship with IL-13 and IL-10 level, symptoms score and quality of life.

**Methods:**

Patients with persistent allergic rhinitis caused by HDM and having symptoms for at least 2 years with or without allergic asthma were involved into the study. Measurements of IL-22, IL-13 and IL-10 and in serum and nasal lavage was performed by ELISA. Questionnaires assessing symptoms severity and quality of life were used.

**Results:**

A tendency was observed that IL-22 in serum and nasal lavage was higher in patients with allergic airway diseases compared to control group (14.86 pg/ml vs. 7.04 pg/ml and 2.67 pg/ml vs. 1.28 pg/ml, respectively). Positive statistically significant correlation was estimated between serum IL-22 and serum IL-10 (rs = 0.57, *p* < 0.01) and IL-13 (rs = 0.44, *p* < 0.05) level. Moreover, positive significant correlation was found between IL-22 in nasal lavage and IL-10 in nasal lavage (rs = 0.37, *p* < 0.05). There was a negative statistically significant correlation between serum IL-22 and Rhinoconjunctivitis Quality of Life Questionnaire (RQLQ) (rs = − 0.42, *p* < 0.05).

**Conclusion:**

Our study showed a possible anti-inflammatory effect of IL-22 in patients with persistent allergic airway diseases caused by HDM.

## Background

Allergic airway diseases—allergic rhinitis and allergic asthma—cause a great burden worldwide [1–5]. The main allergen which is responsible for the persistent allergic rhinitis and allergic asthma is house dust mite (HDM) [6]. The prevalence of allergic airway diseases increases despite modern methods of treatment and better access to them [1, 5, 7]. However, the pathogenesis of these diseases is still under investigation. Allergic rhinitis and allergic asthma are heterogenous diseases and manifest in different phenotypes which may depend on endotypes [1, 8]. Moreover, allergic rhinitis and allergic asthma very often are diagnosed together, that is why the hypothesis of united airway disease was proposed [9, 10].

For better management of allergic airway diseases, it is particularly important to determine its phenotype and endotype. This can ensure personalized approach, treatment options and prognosis for current patient especially when common medications are not effective enough. However, there is no unified classification of phenotypes and endotypes of allergic airway diseases. That is why scientists are still investigating immunologic mechanisms that can be important in the development of allergic rhinitis and allergic asthma.

Cell-mediated effector immunity can be divided in three types: type 1, type 2, and type 3 [11]. It is known that in allergic airway diseases the main role belongs to T lymphocyte helper (Th) 2 producing interleukin (IL) 4, IL-5 and IL-13 (type 2 immunity) [1, 8, 11]. Type 1 immunity consists of Th1 and its produced cytokines such as interferon -γ (IFN -γ) and type 3 immunity consists of Th17 cells producing IL-17 and IL-22 [11]. It is thought that type 1 and type 3 immunity is important in the development of autoimmune disorders or chronic non-allergic airway inflammation [11, 12]. However, there are evidences that IL-22 can also be involved in allergic airway inflammation [13–16].

IL-22 was firstly described in 2000 [17]. Initially it was thought that it is secreted only by Th17, however, later a new T cell subpopulation Th22 was found [18, 19]. Now it is known that IL-22 can be secreted by Th1, Th2, Th17, Th22, natural killers, and innate lymphoid cells [20]. Despite the evidence that IL-22 can be important in the pathogenesis of allergic asthma and allergic rhinitis scientists argue if this cytokine acts as proinflammatory or anti-inflammatory agent [14, 21].

The aim of this study was to investigate IL-22 level in patients with persistent allergic airway diseases caused by HDM in comparison with healthy individuals and to evaluate its relationship with IL-13 and IL-10 level, symptoms score and quality of life.

## Methods

### Study population

Patients with persistent allergic rhinitis diagnosed according to Allergic Rhinitis and its Impact on Asthma (ARIA) and having symptoms for at least 2 years with or without allergic asthma diagnosed according Global Initiative for Asthma (GINA) were involved into the study. The inclusion criteria were hypersensitivity to HDM diagnosed by skin prick test or allergen specific immunoglobulin (Ig) E test, no use of local or systemic glucocorticoids or other immunosuppressant drugs for at least 1 month before the study, no use of antihistamines for 1 week before the study and no respiratory infection for at least 1 month before the study. Exclusion criteria were treatment with allergen immunotherapy, relevant hypersensitivity to other inhaled aeroallergens, malignant diseases and systemic autoimmune or other diseases. Healthy patients were involved into the study as the control group.

Patients additionally were divided into three groups: (1) allergic rhinitis, (2) allergic rhinitis with allergic asthma and (3) control group.

### Questionnaires

Patients with allergic rhinitis were asked to complete Total nasal symptom score (TNSS) [22] and Rhinoconjunctivitis Quality of Life Questionnaire (RQLQ) [23].

Patients with allergic asthma additionally were asked to complete Asthma control test (ACT) [24] and Asthma Quality of Life Questionnaire (AQLQ) [25].

All subjects had to complete Pittsburgh Sleep Quality Index (PSQI) [26].

The permission to use validated questionnaires (Lithuanian versions) was received.

### Evaluation of allergic sensitization

Allergic sensitization was determined by skin prick test or allergen specific IgE test.

Skin prick test was performed according to the standard protocol with standard inhalant allergens (Diater, Spain) on the inner forearm. Drop of different allergen solution was placed at 3 cm distant from each other. Histamine solution 10 mg/ml was used as a positive control and diluent was used as a negative control. The skin was pricked through the drop using the tip of a lancet (separate lancet was used for all allergen drops). Skin reaction was assessed after 15 min. Wheal was measured using ruler. The test was assumed as a ‘positive’ if diameter of the wheal was at least 3 mm.

Measurement of allergen specific IgE was performed using standard immunoblot analysis according to the manufacturer's instructions (Euroimmun, Germany). Total IgE in serum was measured using enzyme immunoassays (AIA-FAC IgEII Tosoh Bioscience, Japan).

### Peripheral blood collection and processing

Peripheral vein puncture was performed for all subjects. Blood samples were drawn into KEDTA tubes for investigation for complete blood count and into serum tubes. Serum tubes were stored at room temperature for 30–60 min. and centrifuged at 3500 rpm for 10 min, and serum was separated and frozen at − 80 °C for further analysis.

### Nasal lavage collection and processing

Nasal lavage was collected for all subjects using 5 ml isotone saline per nostril with reclined neck (about 30 °C from the horizontal) and closed soft palate. After 30 s the subject flexed the neck draining lavage fluid into a sterile vessel. Nasal lavage fluid was frozen at − 80 °C for further analysis.

### Nasal smear for eosinophil detection

Nasal smears of patients were obtained by gently swabbing the nasal inferior turbinate with a cotton-tipped swab. The sample was then placed on a surface of glass microscope slide and stained with Giemsa stain. All specimens were examined by qualified pathologist.

### Laboratory analysis of cytokines

Measurements of IL-22, IL-13, and IL-10 and in serum and nasal lavage was performed by ELISA using commercial kits (Elabscience Biotechnology Inc., USA).

### Statistical methods

Statistical analysis was performed using statistical program SPSS 20. Non-parametric statistical methods were applied.

Mann–Whitney U and Kruskal–Wallis H tests were applied for comparison of variables between patients with allergic airway diseases and healthy individuals, and between patients with allergic rhinitis only, patients with allergic rhinitis and allergic asthma and healthy individuals.

Methods of correlation (Spearman’s coefficient) was used to find associations between IL-22 and IL-13, IL-10, eosinophil count, neutrophil count, lymphocyte count, total IgE, TNSS, ACT, AQLQ, RQLQ and PSQI.

A *p* value of < 0.05 was considered statistically significant.

## Results

### Characteristics of studied subjects

Fourty subjects were involved into the study. Demographic characteristics are presented in Table 1. Subjects’ distribution according to the age and sex did not differ between different groups. Duration of rhinitis symptoms was slightly longer in patients with allergic rhinitis and allergic asthma compared with patients with allergic rhinitis only. Majority of patients with allergic airway diseases caused by HDM had sensitivity to other inhalant allergens.

**Table 1:** Demographic characteristics and responses to questionnaires of study population

	Patients with allergic airway diseases (N = 31)		Control group (n = 9)
	Patients with allergic rhinitis (N = 22)	Patients with allergic rhinitis and allergic asthma (N = 9)	
Male/female, N	10/12	4/5	2/7
Age, years, mean ± SEM	27.27 ± 1.54	29.44 ± 2.99	34.33 ± 4.71
Duration of rhinitis symptoms, years, mean ± SEM	13.33 ± 1.89	16.00 ± 3.84	N/A
Duration of asthma symptoms, years, mean ± SEM	N/A	11.20 ± 5.90	N/A
Hypersensitivity to other aeroallergens, N (%)	15 (75.00)	7 (77.80)	N/A
TNSS, mean ± SEM	4.32 ± 0.55	3.88 ± 0.74	N/A
ACT, mean ± SEM	N/A	19.5 ± 1.10	N/A
RQLQ, mean ± SEM	1.61 ± 0.24	1.48 ± 0.50	N/A
AQLQ, mean ± SEM	N/A	5.62 ± 0.40	N/A
PSQI, mean ± SEM	7.05 ± 0.76	6.50 ± 1.64	5.67 ± 0.62

There were no differences of TNSS, RQLQ and PSQI scores between patients with allergic rhinitis and allergic asthma and patients with allergic rhinitis only (Table 1). PSQI also did not differ significantly between patients with allergic airway diseases and healthy individuals.

### Blood cells and serum IgE

There were no statistically significant differences between leukocytes, neutrophils, lymphocytes, and monocytes count in blood between studied groups (Table 2). Whereas blood eosinophil count and total IgE level in serum were statistically significantly higher in patients with allergic airway diseases than in healthy individuals (Table 2).

**Table 2:** Total blood count, serum total IgE and eosinophils in nasal smear

	Patients with allergic airway diseases (N = 31)	Control group (n = 9)
Leukocytes, × 10/9/l	6.22 ± 0.30	6.19 ± 0.37
Neutrophils × 10/9/l	3.38 ± 0.18	3.78 ± 0.37
Lymphocytes × 10/9/l	2.07 ± 0.17	1.78 ± 0.12
Monocytes × 10/9/l	0.53 ± 0.03	0.46 ± 0.03
Eosinophils × 10/9/l	0.25 ± 0.03*	0.13 ± 0.04
Total IgE, kU/l	269.26 ± 50.60**	34.83 ± 20.26

^*^p < 0.05 compared with control group

^**^p < 0.01 compared with control group

Data are presented as mean ± SEM

### Eosinophils in nasal smear

Eosinophil count in nasal smear did not differ significantly between patients with allergic airway diseases and healthy individuals (2.67 ± 0.64% vs. 0.25 ± 0.16%). However, a tendency was observed that eosinophil count in nasal smear is higher in patients with allergic airway diseases than in subjects from control group (*p* = 0.06).

### Cytokines level in serum and nasal lavage

**Fig. 1:**
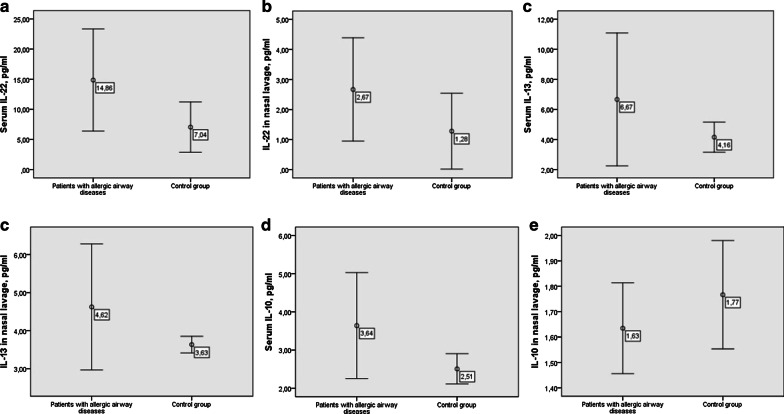
IL-22, IL-10 and IL-13 level in serum and nasal lavage in patients with allergic airway diseases and healthy individuals

No statistically significant differences of IL-22, IL-13 and IL-10 level in serum and nasal lavage were found between patients with allergic airway diseases and control group (Fig. 1). However, a tendency was observed that IL-22 in serum and nasal lavage was higher in patients with allergic airway diseases compared to control group (Fig. 1).

### Correlation between IL-22 and IL-10, IL-13 and scores of symptoms and quality of life

**Fig. 2:**
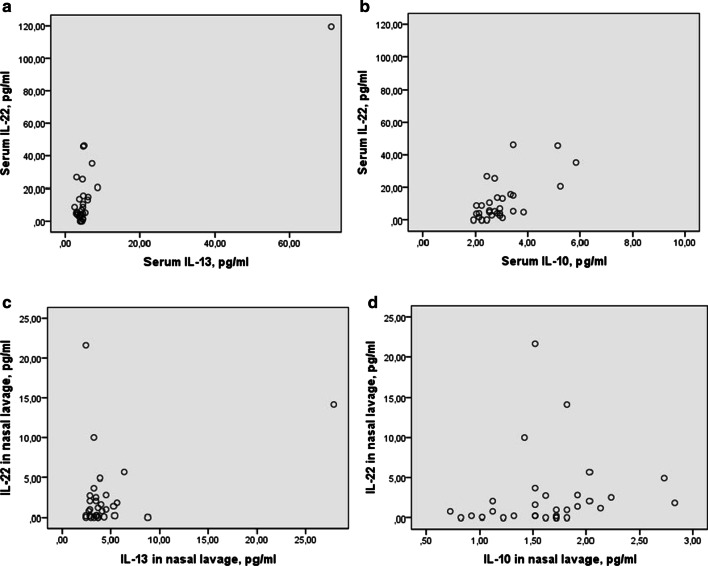
Spearman’s correlation between IL-22, IL-10 and IL-13 in serum and nasal lavage in patients with allergic airway diseases. **a** rs = 0.44, p < 0.05, **b** rs = 0.57, p < 0.01, **c** rs = 0.1, *p* > 0.05, **d** rs = 0.37, p < 0.05

Positive statistically significant correlation was estimated between serum IL-22 and serum IL-10 and IL-13 level (Fig. 2a, b). Moreover, positive significant correlation was found between IL-22 in nasal lavage and IL-10 in nasal lavage (Fig. 2d). However, there was no relation between IL-22 level in nasal lavage and IL-22 level in serum (rs = -0.19, *p* = 0.31).

There was a negative statistically significant correlation between serum IL-22 and RQLQ (Table 3). However, there were no correlations between serum IL-22 level and TNSS, AQLQ and ACT. IL-22 in nasal lavage also did not correlated with any score of used questionnaires (Table 3).

**Table 3:** Spearman’s correlation between IL-22 and TNSS, ACT, RQLQ, AQLQ and PSQI in patients with allergic airway diseases

	Serum IL-22	IL-22 in nasal lavage
TNSS	− 0.22	− 0.12
ACT	0.27	0.08
RQLQ	− 0.42*	0.11
AQLQ	0.66	− 0.15
PSQI	− 0.14	0.14

^*^p < 0.05

There was a negative correlation between leukocytes count in blood and IL-22 level in nasal lavage, however, no other correlations between IL-22 level in serum and nasal lavage and serum total IgE, leukocytes subpopulations in blood and eosinophils in nasal smear (Table 4). IL-22 level in nasal lavage negatively correlated with eosinophil count in nasal smear in patients with allergic rhinitis and allergic asthma (rs = − 0.83, *p* < 0.05). Moreover, in this patients’ group negative correlation was also estimated between IL-22 level in nasal lavage and IL-22 level in serum (rs = − 0.70, *p* < 0.05).

**Table 4:** Spearman’s correlation between IL-22 and inflammatory markers in patients with allergic airway diseases

	Serum IL-22	IL-22 in nasal lavage
Total IgE, kU/l	0.08	− 0.03
Leukocytes, × 10/9/l	0.15	− 0.37*
Neutrophils × 10/9/l	0.12	− 0.30
Lymphocytes × 10/9/l	0.06	− 0.28
Monocytes × 10/9/l	0.12	− 0.32
Eosinophils × 10/9/l	0.11	− 0.20
Eosinophils in nasal smear (%)	− 0.02	− 0.21

^*^p < 0.05

## Discussion

Our study showed a tendency that local and systemic IL-22 level was increased in patients with allergic airway diseases caused by HDM. Both IL-22 level in serum and IL-22 level in nasal lavage were positively associated with IL-10 level in serum and nasal lavage. Our findings support hypothesis of anti-inflammatory effect of IL-22 in persistent allergic airway diseases. However, experimental, and clinical studies provide controversial data about possible role of IL-22 in these diseases [14, 21].

Our findings are in agreement with the majority of clinical studies investigating IL-22 level in patients with allergic rhinitis and/ or allergic asthma and revealed that concentration of IL-22 in serum or plasma was higher in these patients than in healthy subjects [27–32]. Though, studies investigating local IL-22 level provided controversial results. For example, Shahsavan et al. found that the gene expression level of IL-22 in human nasal mucosa was higher in patients with persistent allergic rhinitis than in healthy individuals [28]. In contrary, Andersson et al. analysed IL-22 level in childrens’ with severe asthma bronchoalveolar lavage and did not found differences between asthma group and control group [31]. However, there are more evidence that IL-22 level is higher in patients with allergic airway diseases. Differences between studies can occur due to relatively small studies samples and different methods used for investigation of IL-22 level.

In our study level of IL-22 was positively related with IL-10 despite local or systemic. This supports hypothesis of anti-inflammatory effect of IL-22. We found positive correlation between local and systemic IL-22 level and local and systemic IL-10 level. We did not find any clinical study investigating relationship between IL-22 and other proinflammatory and anti-inflammatory cytokines in allergic airway diseases in PubMed database. But experimental studies revealed that IL-22-positive mice had a lower level of IL-13 in the bronchoalveolar lavage after ovalbumin stimulation in comparison with IL-22-negative mice [33, 34]. Nakagome et al. showed that IL-22-producing plasmid vector that was delivered before the sensitization with ovalbumin suppressed eosinophilic airway inflammation and increased the level of IL-10 [35]. However, other study showed that ovalbumin-challenged and IL-22-deficient mice had low level of IL-5, IL-13 and IL-33 [36]. It is thought that IL-22 plays its role via subsequent Janus kinase-signal transducers and activators of transcription (JAK-STAT) signaling pathways [37].

Our study showed that IL-22 level in nasal lavage negatively correlated with eosinophil count in nasal smear in patients with allergic rhinitis and allergic asthma. In contrary, Shahsavan et al. revealed positive link between serum IL-22 level and eosinophil count in nasal mucosa [28]. However, IL-22 level in serum may not reflect the local IL-22 level and this could be the reason for controversial data. Some experimental studies showed that ovalbumin-challenged and IL-22-deficient mice had a low level of eosinophils in bronchoalveolar lavage and lung tissue and reduced leukocyte infiltration [36]. In contrary, some other studies with animals reported that after ovalbumin stimulation IL-22-positive mice had a reduced number of eosinophils in the bronchoalveolar lavage when compared with IL-22-negative mice [33, 34]. Moreover, Besnard et al. suggested that IL-22 may play dual role in allergic airway inflammation—it may be necessary for the induction of inflammation, but during continuous allergen challenge, the lack of IL-22 may exacerbate allergic inflammation [36]. In contrary, Nakagome et al. presented that IL-22 could have an immunosuppressive effect during the early stage of an immune response by reducing eosinophilic airway inflammation [35]. The differences between the results from different studies could be explained by different conditions and methods of experiments.

We did not find significant correlation between IL-22 in serum and IL-22 in nasal lavage. Interestingly, we found negative correlation between serum IL-22 level and IL-22 level in nasal lavage only in patients with allergic rhinitis together with allergic asthma. These findings suggest that systemic IL-22 level may not reveal local IL-22 concentration. We did not find other studies investigating relation between local and systemic IL-22 level in allergic airway diseases.

Our study revealed a negative link between serum IL-22 and RQLQ in patients with allergic airway diseases. In contrast, other two studies performed by Shahsavan et al. and Zhu et al. revealed positive relationship between serum IL-22 and rhinitis and asthma symptoms scores in patients with allergic rhinitis and allergic asthma [28, 30]. Farfariello et al. found that IL-22 was higher in patients with severe allergic rhinitis and severe allergic asthma than in patients with moderate allergic rhinitis and allergic asthma [29]. Zhao et al. also found that level of IL-22 in plasma was consistently increased with the severity of asthma [32]. Moreover, Sherkat et al. showed a significantly higher level of IL-22 in serum and sputum samples from adults with severe asthma in comparison to patients with moderate asthma [38]. These findings show that IL-22 depends on the disease severity. In contrast, Bayrak Degirmenci et al. did not find any relation between plasma IL-22 and symptoms score in patients with allergic rhinitis [27]. As mentioned before, these differences between studies could be determined by relatively small studies samples, different methods of IL-22 investigation and use of different tools for investigating severity of symptoms.

To our knowledge there are no published articles about IL-22 level in nasal lavage and relation between systemic and local IL-22 level and other cytokines. Moreover, there are lack of articles presenting data about IL-22 link with symptoms score, quality of life and different inflammatory markers in patients with allergic airway diseases.

## Conclusions

Our study showed a possible anti-inflammatory effect of IL-22 in patients with persistent allergic airway diseases caused by HDM.

## Data Availability

All data generated or analysed during this study are included in this published article.

## Abbreviations

IL: Interleukin
HDM: House dust mite
Th: T lymphocyte helper
ARIA: Allergic Rhinitis and its Impact on Asthma
GINA: Global Initiative for Asthma
Ig: Immunoglobulin
TNSS: Total nasal symptom score
ACT: Asthma control test
RQLQ: Rhinoconjunctivitis Quality of Life Questionnaire
AQLQ: Asthma Quality of Life Questionnaire
PSQI: Pittsburgh Sleep Quality Index
